# Supplementary material for: The RNA secondary structure of androgen receptor-FL and V7 transcripts reveals novel regulatory regions
Source: Nucleic Acids Res. 2024 Mar 30;52(11):6596–613. doi: 10.1093/nar/gkae220 (PMC11194067; doi:10.1093/nar/gkae220)
Supplement: gkae220_Supplemental_Files [file gkae220_supplemental_files.zip › Supplementary File S5.zip!/Supplementary File S5/FL_CaCo.zip!/FL_CaCo/AR_M60_1.fold.R2R.sto.pdf]

AR_M60_1

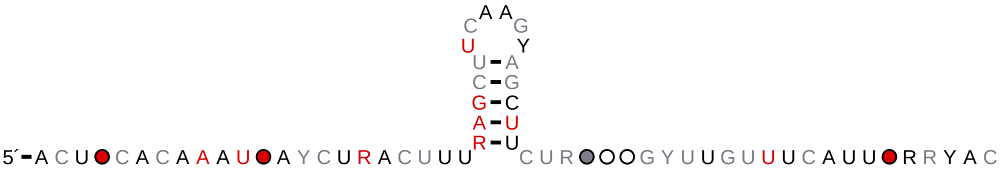
5´
A C U C A C A A A U A Y C U R A C U U U
R A G C U U C
C A A G
Y A G C U U
C U R G Y U U G U U U C A U U R R Y A C
